# How Anti-Substance Abuse Campaigns Influence Substance Abusers’ Psychological Health in Chinese Communities: The Mediating Role of Perceived Stigma

**DOI:** 10.3390/ijerph19116687

**Published:** 2022-05-30

**Authors:** Yonghui Zeng, Li Han, Yu Cheng, Cindy Xinshan Jia

**Affiliations:** 1 School of Economics and Statistics, Guangzhou University, Guangzhou 510006, China; yonghuizeng@scau.edu.cn; 2 Department of Social Work, School of Public Administration, South China Agricultural University, Guangzhou 510640, China; lynn_han1216@scau.edu.cn; 3 School of Education Science and Law, Xiangnan University, Chenzhou 423043, China; 4 School of Sociology and Anthropology, Sun Yat-sen University, Guangzhou 510275, China

**Keywords:** substance abusers, perceived stigma, psychological health

## Abstract

The current study explored how anti-substance abuse campaigns influence substance abusers’ psychological health through the perception of stigma. The study is based on a sample of substance abusers who received community-based treatments (*n* = 3457) and used structural equation modeling to estimate the role of perceived stigma in mediating between perceptions of overstatement of harm conveyed in anti-substance abuse campaigns and psychological outcomes. The results revealed that substance abusers’ perception of overstatement of the harm caused by the substances and substance abusers enhanced their perceived stigma and impaired their psychological health in terms of anxiety, depression, and somatization, through both direct and indirect pathways. The results advocate for proper strategies in the design of anti-substance abuse campaigns. Possible initiatives to reduce substance abusers’ perceived stigma are recommended.

## 1. Introduction

Stigma is a robust correlate of psychological health [1]. Referring to a “mark” or an attribute that defines an individual based on stereotyping, stigma leads to discriminatory behavior against individuals within the inferior stereotyped groups [2,3,4]. Substance abusers frequently face public disapproval due to pre-existing stereotypes, such as being dangerous and irresponsible [5,6]. Substance abusers who are aware of such stereotypes may engage in personal devaluation and internalize these stigmas [7]. As a result, stigma damages several aspects of a substance abuser’s life, including making their access to resources more unequal, diminishing their willingness to seek help, limiting their opportunities for recovery, and creating poor health outcomes [5,8,9].

Anti-substance abuse campaigns are one of the sources of such stigma, e.g., [10]. Although such campaigns have been designed to protect the majority of non-users from substance abuse, the information conveyed in these campaigns usually employs “scare tactics” and defines substance abusers as a “social evil” [11]. Along with harsh criminal sentences for using illegal substances, in some countries anti-substance abuse campaigns appear to further label substance abusers as socially excluded and create stereotypes [11,12]. This social exclusion and discrimination are also risk factors for substance abusers’ psychological health, such as causing depression [13]. Nevertheless, only a few studies have explored anti-substance abuse campaigns’ negative influences, e.g., [14]; how anti-substance abuse campaigns negatively influence substance abusers’ well-being has rarely been examined, e.g., [11].

As a conceptual form of stigma, perceived stigma refers to an individual’s beliefs and perceptions regarding the prevalence of stigmatized attitudes and behaviors that others hold toward them [15]. Perceived stigma is significantly influenced by policy [11], public discourse [10], enacted stigma [16], treatment [17], types of stereotyped behaviors or symptoms [18,19], social supports [20], demographic characteristics, and other factors. It further enhances internalized stigma [15], generates a lower self-concept [6], decreases psychological functioning such as empathy [21] and shame [22], increases mental disorders such as depression [6,15,23], and reduces treatment-seeking behaviors [24] and treatment outcomes [25,26].

Perceived stigma rationally serves as a mediator between perceptions of overstatement of harm conveyed in anti-substance abuse campaigns and psychological symptoms among substance abusers. According to the process of stigma internalization, with pre-existing social categorizations (i.e., stereotypes) shaped by public discourse, substance abusers will gradually see themselves as being part of the stereotyped inferior group and will consistently enact stigma experiences [27], subsequently leading to possible experiential avoidance [28]. Thus, with exposure to the anti-substance abuse messages that are conveyed in anti-substance abuse campaigns, substance abusers perceive a social categorization and identity confirmation with the inferior stereotyped group, which rationally causes avoidance [29], thought suppression [30], and subsequent psychological symptoms such as depression [31]. Nevertheless, how anti-substance abuse campaigns negatively influence substance abusers’ psychological health through perceived stigma remains overlooked.

China has a large population of registered substance abusers who suffer from stigma [32,33,34,35]. According to a report by the China National Narcotic Control Commission (CNNCC), the number of substance abusers, defined as individuals who have been medically diagnosed with an addictive disorder and are registered in an administrative system for substance abusers, reached 1.8 million in 2020. In China, there are four types of substance abuse treatment programs: voluntary rehabilitation, compulsory rehabilitation, community detoxification, and community rehabilitation [36]. Voluntary rehabilitation is one in which the substance abuser participates on a voluntary basis. For substance abusers with any history of arrest or a criminal record, the other three types of compulsory programs are required by the country’s law enforcement agencies [37]. For those whose substance abuse is severe, an institutional compulsory rehabilitation is required, which includes two years of physical recovery, psychological education, and abstinence. For minor substance users and individuals who have completed the compulsory rehabilitation, community detoxification and community rehabilitation (CDCR) are required. CDCR usually lasts no more than three years and includes an individualized plan, facilitated by local community administrators and social workers, and with psychological education and counseling related to health, family relationships, and employment. Community detoxification focuses more on treating the addiction, whilst community rehabilitation emphasizes community reentry. CDCR is one of the most important procedures for substance abusers’ recovery [34]. Nevertheless, the existing literature has focused on the effects of pharmaceutical treatment outcomes, such as the rehabilitation effects of Methadone Maintenance Treatment e.g., [32,38,39]. The impact of non-pharmaceutical programs, especially on the stigma associated with Chinese substance abusers, has barely been investigated [40,41].

There are several variables that contribute to the stigma that Chinese substance abusers experience. As registration with a substance-abuse system is viewed as a lifelong “stamp” on substance abusers [42,43], China’s policy on registering substance abusers exacerbates the internalization of stigma, structurally labeling them as an inferior group. In addition, the drug policy used to view substance abusers as offenders and the corresponding anti-drug campaigns that have been implemented [44,45], including conveying “scare tactic” messages in the communities that emphasize the severe harmfulness and related harsh punishment brought about by the abuse of substances. Although the current policy has begun to treat substance abusers as victims, the ongoing anti-substance abuse campaigns implemented in the communities, with the aim to prevent substance abuse among non-users in the neighborhoods, are still using the same scare tactics [46]. There are usually two types of “scare tactic” messages. The first type of “scare tactics” message focuses on the potential harm caused by substance abusers, such as “if one gets addicted, the entire family would be destroyed.” The second type aims at the potential harm caused by the addictive substances, such as “choosing addictive substances is choosing death”. These “scare tactic” messages are not surprisingly overstating the harmfulness, which rationally enhances perceived stigma among substance abusers. Furthermore, Chinese society is culturally a collectively-oriented one in which individuals tend to emphasize social relationships. As such, the social devaluation that causes stigma is particularly harmful to substance abusers’ well-being [12,47,48]. CDCR substance abusers, who are residentially treated in their communities, are exposed to anti-substance abuse campaigns there, and experience stigma from their families, friends, professional groups, and society, all of whom also have been exposed to anti-substance abuse messages [34,49,50,51]. Thus, CDCR substance abusers might be particularly vulnerable to stigma depending on their treatment setting [26]. Consequently, such stigma is associated with dropping out of treatment programs [52] and a higher risk of unemployment [43], as well as possible psychological symptoms [53]. However, how these anti-substance abuse messages influence CDCR substance abusers’ psychological outcomes remains largely unknown in the Chinese context.

The current study explores CDCR substance abusers’ psychological outcomes, particularly the influence of community anti-substance abuse messages and perception of stigma. According to the above discussions, there are two types of overstatement of harm conveyed in anti-substance abuse campaigns, one is associated with substance abusers (i.e., perceived overstatement of the harm-substance abusers, POH-SA), and the other is associated with the substances themselves (i.e., perceived overstatement of the harm-substances, POH-S). According to the relationships between enacted stigma related experiences and psychological symptoms e.g., [13,16,49], we hypothesized that:

**Hypothesis** **1** **(H1).** *POH-SA positively influences psychological symptoms*.

**Hypothesis** **2** **(H2).** *POH-S positively influences psychological symptoms*.

Furthermore, CDCR substance abusers’ perceived stigma should be influenced by these two types of perceived overstatement of the harm conveyed in community anti-substance abuse campaigns, and their perceived stigma further influences their psychological health, e.g., [8,16,25]. We hypothesize that:

**Hypothesis** **3** **(H3).** *Perceived stigma positively mediates the effects of the POH-SA and POH-S on psychological symptoms*.

## 2. Materials and Methods

### 2.1. Data and Sample

Ethical approval for this study was obtained from the Institutional Review Board (IRB) of the Anthropology Department at Sun Yat-sen University in Guangzhou, China. A cross-sectional design with two-stage cluster sampling was employed. First, using “city” as the sampling unit, eight cities in Guangdong Province were randomly selected. Second, using “street” as the sampling unit, fifteen street-level units were randomly selected per city. Then, ten to fifty individuals who received any type of substance abuse treatment were randomly referred by two province-level agencies to participate in the survey.

Online questionnaires were used on a voluntary basis. Only those who agreed with the informed consent on the first page proceeded to answer the questionnaire. A total of 6128 responses were collected, and 3457 were maintained after screening for non-CDRC participants and duplicated responses.

### 2.2. Measurement

#### 2.2.1. Dependent Variables

Psychological symptoms. As psychological symptoms are associated with stigma among individuals with substance abuse [54], anxiety, depression, and somatization were measured by the Brief Symptom Inventory 18 (BSI-18) [55]. Respondents rated on a 5-point frequency scale their experiences of psychological symptoms in the previous week, from “not at all” = 0 to “very frequently” = 4. Composite scores for three subscales were achieved with good internal consistency (i.e., Cronbach’s α s of 0.92, 0.93, and 0.92 on anxiety, depression, and somatization, respectively).

#### 2.2.2. Independent Variables

Perceived stigma. As mentioned above, stigma impacts people at various social levels [26,50]. The survey focused on the most influential levels of family, society, and official propaganda, and asked about the degree to which respondents perceive stigmatizing attitudes and actions from these three sources. Responses were originally rated on a 4-point scale from “not at all” = 1 to “very much” = 4. The four levels yielded a solid internal consistency (i.e., Cronbach’s α = 0.85), and the composite score was computed.

Perceived overstatement of the harm-substance abusers (POH-SA). A single item statement of “anti-substance abuse campaigns are overstating the harm caused by substance abusers” was used to ask about participants’ degree of perception. It was rated on a 4-point scale from “not at all” = 1 to “very much” = 4.

Perceived overstatement of the harm-substances (POH-S). A single item statement of “anti-substance abuse campaigns are overstating the harm caused by substances” was used to ask about participants’ degree of perception. It was rated on a 4-point scale from “not at all” = 1 to “very much” = 4.

#### 2.2.3. Controlled Covariates

Type of substances. The type of substance that is abused influences the stigma process [56]. According to the substance usage representativeness in the current sample, the substances were classified as one of three types: heroin, methamphetamine, and other. Other substances include opium, cocaine, cannabis, morphine, ketamine, ecstasy, and other psychoactive drugs. The categories were dummy-coded as a history of usage = 1 and no history of usage = 0.

Duration of abstinence. The duration of abstinence was controlled, as it influences stigma [57]. The participants were asked about their estimated number of days of abstinence. As the participants on average had a three-month abstinence period (SD = 1.03), days were transferred into months for analysis.

Physical unhealth. Physical health status was controlled, as it is a robust correlate of psychological symptoms [58]. It was measured by a single question: “how many days did you feel physically unwell in the past week?” Participants reported the number of days, with a larger number representing poorer self-reported physical health status.

Demographic characteristics. Respondents were asked about demographic factors including employment status, gender, education, age, income, and marital status [53,59,60]. Respondents’ employment status was measured by a single question asking about the title of their current job. A score of 1 was given if the respondent reported having a steady job, and 0 was given if the respondent reported being unemployed, in the process of looking for a job, or staying at home. Gender was coded as “male” = 1 and “female” = 0. Education was coded into years of education as “Primary school or below” = 9, “High school/vocational school” = 12, “College degree/Diploma” = 15, “Undergraduate education/Bachelor” = 16, and “Postgraduate education/Master or above” = 19. Age was coded into years of age. Income was coded according to respondents’ personal monthly income. Furthermore, as there was a limited number of respondents who were divorced or had a marital status other than married (1.18%), marital status was coded as “married” = 1 and “other” = 0. A blank report was coded as a missing value.

### 2.3. Data Analysis

We employed Stata 15.0 (StataCorp LLC, College Station, TX, USA) for zero-order correlations and Structure Equation Modeling (SEM) with Maximum Likelihood estimation. As the current study aimed to understand how stigma influences general psychological symptoms, the psychological symptoms of anxiety, depression, and somatization were modeled as a latent variable to capture the shared variance [61]. The indirect effects of mediation were examined using the Monte Carlo method, as that method was preferred for SEM [62,63]. As the proportions of missing data were smaller than 0.01% among all key variables, the missing data was auto-deleted in the analyses.

## 3. Results

Regarding the missing data, observations with blank responses were deleted. Key variables reported a low percentage of missing values (i.e., less than 0.01%). Thus, the missing data was auto-deleted in the analyses. Common method bias was checked using the Harman Single Factor technique [64]. All research items exacted one factor that accounts for 39.73% of the total variance, indicating no common method bias.

Sample demographic statistics were presented in Table 1. The current sample of community substance abusers was predominantly male (i.e., 91.29%), with an average age of 37.82. They generally had a secondary school education (i.e., 8.56 years of education) and a low level of monthly income (i.e., M = 2.72, SD = 1.61). Over half of the sample was married (i.e., 55.51%) and 75.12% were currently employed. Regarding the substance type, heroin accounted for one-third, methamphetamine accounted for half, and other types of substances accounted for one-sixth of the sample.

The zero-order correlations between independent variables, mediators, and dependent variables are presented in Table 2. As expected, the three dimensions of psychical symptoms were strongly inter-correlated (i.e., r = 0.84–0.88, *p* < 0.00). Perceived stigma was slightly correlated with psychological symptoms (i.e., r = 0.18, *p* < 0.00), and, as expected, strongly correlated with the POH-SA (i.e., r = 0.86, *p* < 0.00). It failed to show any inter-correlations between the two types of perception of overstatement of harmful, or between the perceived stigma and POH-S.

**Figure 1:**
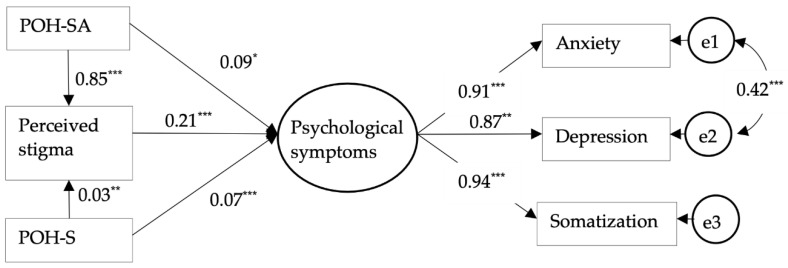
The standardized SEM results. Note. *** *p* < 0.00, ** *p* < 0.01, * *p* < 0.05. POH-SA = Perceived overstatement of the harm-substance abusers; POH-S = Perceived overstatement of the harm-substances.

The SEM results are shown in Figure 1 and Table 3. According to SEM model fit standards [65,66], the current model yielded a good model fit: ${Χ}^{2}/df=$ 3.57, which falls within the range 2–5. The comparative fit index (CFI) = 0.99 > 0.90, the Tucker–Lewis index (TLI) = 0.98 > 0.90, and the root mean square error of approximation (RMSEA) = 0.03 < 0.05. According to the cutoff values of standardized SEM coefficient [67] p. 208, H1 and H2 were supported in that POH-SA (β=0.09, p<0.05) and POH-S (β=0.07, p<0.00) positively influenced psychological symptoms with a small effect size. Regarding H3, POH-SA (β=0.85, p<0.00) and POH-S (β=0.03, p<0.01) positively influenced perceived stigma, whilst perceived stigma positively influenced psychological symptoms (β=0.21, p<0.00). Second, according to the cutoff values of the mediation effect size [68], the Monte Carlo method (5000 replications) showed the indirect effects on psychological symptoms of the perceived stigma, mediating the influence of POH-SA in a medium to large effect size (b=0.18, SE=0.03, 95% CI=0.12 ~ 0.24, p<0.00) and POH-S to a small effect size (b=0.01, SE=0.00, 95% CI=0.00 ~ 0.01, p<0.05). Thus, H3 was also supported.

## 4. Discussion

Stigma serves as one of the most important barriers to recovery for substance abusers [25]. The current study is one of the first to test the negative influences caused by anti-substance abuse campaigns on substance abusers’ psychological health through perceived stigma in Chinese communities. Our findings suggest that a perceived overstatement of the harm in anti-substance abuse campaigns, especially messages related to the substance abusers themselves, greatly enhanced substance abusers’ perception of stigma and reduced their psychological health in terms of anxiety, depression, and somatization. The findings have several implications for research and community healthcare practices.

First, the current study elaborates on the negative effects anti-substance abuse campaigns have on substance abusers’ stigma. Anti-substance abuse campaigns usually have been designed to prevent substance abuse among nonusers, especially those who are vulnerable [14]. To enhance the influence of such campaigns, “scare tactics” that appeal to people’s sense of fear have been a very common approach, e.g., [69]. Regarding the influences of such campaigns on substance abusers, the current findings echo that the public discourse shapes ideas about substance abusers as a “social evil” [10,11]. These anti-substance abuse messages, especially those that overstate the harm caused by substance abusers, negatively influence substance abusers’ perceptions of stigma against them. In comparison, the negative influences of stigma brought about by the perceived overstatement of harm caused by the substances per se was much smaller than the negative influences of the harm resulted from perceptions related to substance abusers. Such findings coincide with the view that stigma that is transmitted through persuasive messages may evoke personal defenses when conflicting with one’s own moral identity [70]. This may be because information related to substance abusers contains greater internal attribution [71], whilst information related only to the substances appears less threatening as it does not involve internal attribution [72]. Future studies may further explore the attribution and persuasion mechanism in substance abusers’ perceptions of anti-substance abuse messages.

Second, as psychological health is one of the most vital outcome variables among substance abusers, we find that substance abusers’ perceptions about overstatement of the harm that is conveyed through anti-substance campaigns has a direct and indirect influence on substance abusers’ psychological symptoms. Moreover, the indirect effect brought about by the perceived overstatement of the harm caused by substance abusers, mediated by the perceived stigma, showed a medium to large effect size. This finding adds to existing knowledge regarding substance abusers’ psychological health, namely, that besides factors such as socio-economic status, informal social supports, substance use history, treatment, and personal dispositions [16,40,42,53,73], perceptions of overstatement of the harm and stigma also matter. It is noteworthy that the current sample only includes CDCR substance abusers. As anti-substance abuse campaigns are usually launched in community settings, abusers are exposed to more anti-substance abuse messages than other types of treatment. Previous findings also suggested the benefits of no perceived stigma in the CDCR treatment as compared to other types of treatment [26]. Possible interventions reducing the risks of stigma, such as acceptance and commitment therapy and cognitive behavioral therapy, should be seriously considered in CDCR treatment [74,75]. Furthermore, possible studies may consider comparing the influences of anti-substance abuse messages and their perceived stigma on psychological health in different treatment settings.

Third, the general effects of anti-substance abuse campaigns are worth discussing. Regarding the general population, the effectiveness of prevention among non-users has long been debated (e.g., see the boomerang effect) [14,76]. The current study further implies that such campaigns may have negative influences on substances abusers’ recovery. Therefore, the design of such campaigns is vital [77,78], implying that further studies could explore proper ways of persuading non-users but with minimal side effects on substance abusers. Furthermore, the current study highlights that such anti-substance abuse messages should not target people and their social relationships. A narrow focus on the harm associated with using substances may cause fewer side effects regarding substance abusers’ stigma and psychological health. Such results expand the literature on substance abuse treatment in mainland China, especially using CDCR substance abusers as the target population. Nevertheless, assessments of the effects of anti-substance abuse campaigns in China are still rare, thus calling for future exploration.

The current study has several limitations. First, it employed a self-reporting questionnaire survey in a voluntary manner, which could lead to a possible response bias, such as self-selection and the tendency toward social desirability. Second, although the SEM method was employed, the study was in a cross-sectional design, which cannot fully justify the causal directions. Further investigations through a longitudinal or experimental design would be necessary to help better understand the proposed causal relationships. Third, given the small effect size found in the results, several measurements would need possible improvements in using well-tested scales, such as the perceived stigma scale [15] and rigorous measurements of perceptions of anti-substance abuse messages. Finally, we only focused on substance abusers involved in the community-based treatment programs, whereas future studies may consider using the full sample or other samples to examine factors influencing the possibilities of entering the community-based treatment program or the institutional program, or other cross-program comparisons.

## 5. Conclusions

The current study advanced our understanding of how anti-substance abuse campaigns influence substance abusers’ psychological health through their perceptions of stigma. Using a sample of substance abusers from community-based treatment programs in China’s Guangdong Province (*n* = 3457), the findings revealed that substance abusers’ perception of overstatement of the harm in anti-substance abuse campaigns enhanced their perceived stigma and had negative influences, though slight, on their psychological health. It implies that strategies should be carefully applied in designing anti-substance abuse campaigns, and that the possible protection of substance abusers should also be considered by community governance so as not to associate an overstatement of the harm with the identity of substance abusers. Community health workers should also consider being involved in interventions to reduce the perceived stigma among substance abusers.

## Figures and Tables

**Table 1:** Sample descriptions.

	Female	Male	Total
N	301	3156	3457
	(8.71%)	(91.29%)	(100.00%)
N of employed	210	2387	2597
	(69.77%)	(75.63%)	(75.12%)
N of married	137	1771	1908
	(45.97%)	(56.42%)	(55.51%)
N of using Heroin	52	1042	1094
	(17.28%)	(33.02%)	(31.56%)
N of using Methamphetamine	182	1712	1894
	(60.47%)	(54.25%)	(54.65%)
N of using other types of substances	42	455	497
	(14.24%)	(14.67%)	(14.34%)
M physical unhealth (days per week)	0.55	0.64	0.63
	(0.08)	(0.03)	(1.48)
M of personal monthly income (1000 CNY)	2.37	2.75	2.72
	(0.10)	(0.03)	(1.61)
M of abstinence duration (months)	3.26	3.29	3.29
	(0.06)	(0.02)	(1.04)
M of years of education	8.76	8.54	8.56
	(0.17)	(0.05)	(2.80)
M of years of age	34.38	38.15	37.82
	(0.50)	(0.16)	(9.07)

Note. Percentages of the category total and standardized errors are in parentheses. CNY = Chinese Yuan (1000 CNY ≈ 157 USD). N = number, and M = mean value.

**Table 2:** Zero-order correlational matrix.

	1	2	3	4	5	6
1. Anxiety	—					
2. Depression	0.88 ***	—				
3. Somatization	0.87 ***	0.84 ***	—			
4. Perceived stigma	0.18 ***	0.18 ***	0.18 ***	—		
5. POH-SA	0.14 ***	0.13 ***	0.13 ***	0.86 ***	—	
6. POH-S	0.07 ***	0.08 ***	0.09 ***	0.01	−0.01	—

Note. *** *p* < 0.00. POH-SA = Perceived overstatement of the harm-substance abusers; POH-S = Perceived overstatement of the harm-substances.

**Table 3:** Standardized SEM results (*n* = 2759).

Variables	→POH-SA	→POH-S		→Perceived Stigma		→Psychological Symptoms	
POH-SA				0.85 ***		0.09 *	
POH-S				0.03 **		0.07 ***	
Perceived stigma						0.21 ***	
Controlled Covariates							
Type of substance							
(Base = no usage)							
-Heroin	−0.03	0.03		0.01		−0.01	
-Methamphetamines	0.06 **	0.06 **		0.02 *		−0.01	
-Other	0.00	0.11 ***		0.01		0.06 **	
Abstinence (months)	−0.06 ***	0.02		−0.03 **		−0.05 **	
Physical unhealth	0.06 **	0.04		0.01		0.34 ***	
Demographics							
Employment status	−0.03	−0.05 *		0.03 **		−0.07 **	
Monthly income (CNY)	−0.03	0.05 *		−0.04 **		−0.07 ***	
Male (Base = female)	0.02	0.01		0.02		−0.06	
Married (Base = others)	0.06 **	−0.04		−0.04		0.03	
Age (years)	−0.02	0.06 **		0.03 *		0.04	
Education (years)	0.01	0.01		−0.01		−0.02	
Factor loadings for indicators of the latent variable							
Psychological symptoms ->	Anxiety		Depression		Somatization		
Factor Loading	0.91 ***		0.87 ***		0.94 ***		
Model-fit index							
$\chi^{2}/df$ = 3.57; CFI = 0.99; TLI = 0.98; RMSEA = 0.03, with 90% CI = [0.02–0.04]							

Note. *** *p* < 0.00, ** *p* < 0.01, * *p* < 0.05. POH-SA = Perceived overstatement of the harm-substance abusers; POH-S = Perceived overstatement of the harm-substances.

## Data Availability

The data presented in this study are available upon request from the corresponding author.
